# Diagnostic, Prognostic, and Therapeutic Value of Droplet Digital PCR (ddPCR) in COVID-19 Patients: A Systematic Review

**DOI:** 10.3390/jcm10235712

**Published:** 2021-12-06

**Authors:** Angela Ishak, Mousa M. AlRawashdeh, Stepan M. Esagian, Ilias P. Nikas

**Affiliations:** 1 School of Medicine, European University Cyprus, Nicosia 2404, Cyprus; angela.ishak.10@gmail.com (A.I.); mousa99mahmoud@gmail.com (M.M.A.); 2 Jacobi Medical Center, Department of Medicine, Albert Einstein College of Medicine, The Bronx, New York, NY 10461, USA; esagians@nychhc.org

**Keywords:** quantitative reverse transcription PCR (RT-qPCR), SARS-CoV-2, molecular diagnostic techniques, pathology, clinical, COVID-19 testing, therapeutics, prognosis, survival analysis, evidence-based medicine, public health

## Abstract

Accurate detection of SARS-CoV-2, the pathogen causing the global pandemic of COVID-19, is essential for disease surveillance and control. Quantitative reverse transcription PCR (RT-qPCR) is considered the reference standard test for the diagnosis of SARS-CoV-2 by the World Health Organization and Centers for Disease Control and Prevention. However, its limitations are a prompt for a more accurate assay to detect SARS-CoV-2, quantify its levels, and assess the prognosis. This article aimed to systematically review the literature and assess the diagnostic performance of droplet digital PCR (ddPCR), also to evaluate its potential role in prognosis and management of COVID-19 patients. PubMed and Scopus databases were searched to identify relevant articles published until 13 July 2021. An additional PubMed search was performed on 21 October 2021. Data from the 39 eligible studies were extracted and an overall 3651 samples from 2825 patients and 145 controls were used for our qualitative analysis. Most studies reported ddPCR was more accurate than RT-qPCR in detecting and quantifying SARS-CoV-2 levels, especially in patients with low viral loads. ddPCR was also found highly effective in quantifying SARS-CoV-2 RNAemia levels in hospitalized patients, monitoring their disease course, and predicting their response to therapy. These findings suggest ddPCR could serve as a complement or alternative SARS-CoV-2 tool with emerging diagnostic, prognostic, and therapeutic value, especially in hospital settings. Additional research is still needed to standardize its laboratory protocols, also to accurately assess its role in monitoring COVID-19 therapy response and in identifying SARS-CoV-2 emerging variants.

## 1. Introduction

The coronavirus disease 2019 (COVID-19)—first emerging in Wuhan, China, and rapidly spreading worldwide since then—was declared a global pandemic in 2020 by the World Health Organization (WHO) [1]. COVID-19 is caused by the severe acute respiratory syndrome coronavirus 2 (SARS-CoV-2). Prompt and accurate SARS-CoV-2 detection is the first step towards efficiently controlling this pandemic [2]. Policymakers also rely on accurate SARS-CoV-2 diagnosis to shape the appropriate response to the COVID-19 pandemic [3]. In addition, evidence suggests SARS-CoV-2 viral loads fluctuate through a patient’s hospital course [4], while higher SARS-CoV-2 levels have been linked to increased disease severity and mortality [5]. Furthermore, the levels of SARS-CoV-2 viral load have strongly been associated with its transmission rate in both vaccinated and unvaccinated individuals [6]. Notably, viral load levels can be used to predict the response to treatment in hospitalized COVID-19 patients [7]. This highlights the need for tools that can both accurately diagnose COVID-19, besides quantifying its levels to assess prognosis and monitor therapy response.

Nucleic acid amplification testing (NAAT) by quantitative reverse transcription PCR (RT-qPCR) is considered the reference standard for the detection of SARS-CoV-2, as recommended by the WHO and Centers for Disease Control and Prevention (CDC) [8,9]. However, RT-qPCR testing does have its limitations, including suboptimal sensitivity in low viral concentration samples, susceptibility to PCR inhibitors, false-positive results due to the background DNA/RNA contamination, and the need for a standard curve to quantify the results [10,11]. Of interest, it could even be initially negative in patients with clinical or radiologic suspicion of pneumonia, while being able to detect the viral RNA some days later [12]. Finally, RT-qPCR’s sensitivity varies widely according to the type of samples used [13]. Consequently, there is an emerging need for a more accurate tool to detect SARS-CoV-2 and quantify its levels.

Except RT-qPCR, other approved diagnostic tests include antigen and antibody testing, using flow type (LFA) or enzyme-linked immunoassays (ELISA) [14]. There is also a variety of novel diagnostic techniques, such as clustered regularly interspaced short palindromic repeats (CRISPR), loop mediated isothermal amplification (LAMP), next generation sequencing (NGS), and digital PCR [15]. The latter is an innovative technology able to detect and quantify nucleic acids without needing a standard curve [16,17]. Digital PCR could be either chip-based or more often droplet-based (droplet digital PCR; ddPCR) [18]. There is evidence that ddPCR can play an important role in diagnosing various fungal, bacterial, and viral pathogens with high sensitivity and precision [17]. Studies have shown that ddPCR accurately detects and quantifies SARS-CoV-2, even in low viral load samples; it is also more tolerant to inhibition compared to RT-qPCR [10,19]. This is because PCR inhibitors are diluted following the compartmentalization of the initial PCR reaction mixture into multiple reactions before the amplification step [16,17].

The aim of this systematic review was to collect all available literature and qualitatively evaluate ddPCR’s diagnostic, prognostic, and therapeutic value in COVID-19 patients. Our hypothesis was that ddPCR exhibits an enhanced ability to detect the SARS-CoV-2 RNA than traditional testing such as RT-qPCR, while its accurate RNA quantification could have significant prognostic and therapeutic value. To our knowledge, this is the first systematic review that jointly assesses the diagnostic and prognostic role of ddPCR in COVID-19 disease.

## 2. Results

### 2.1. Literature Search

**Figure 1:**
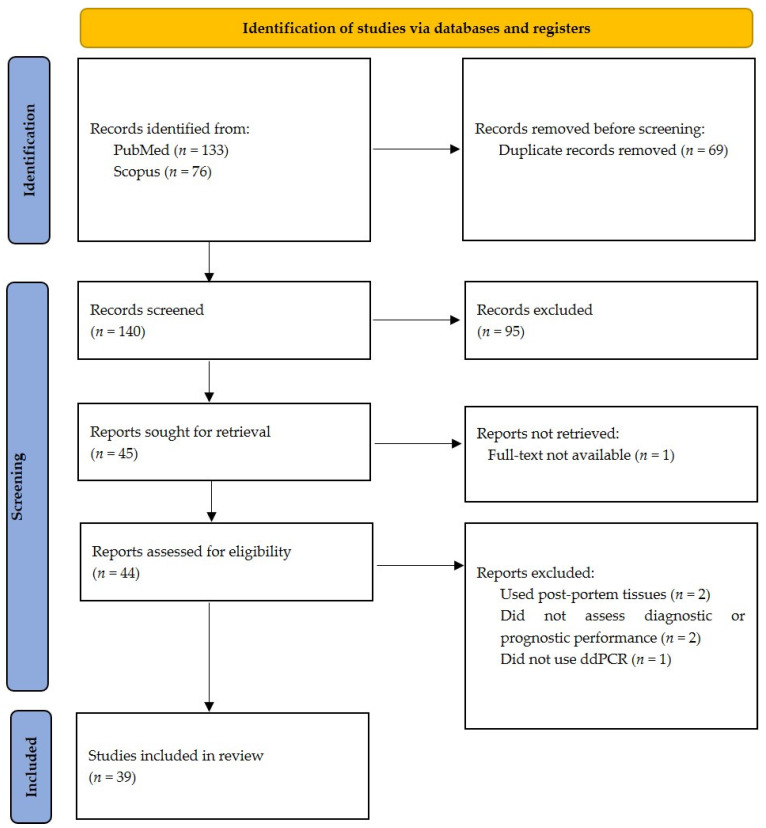
PRISMA flowchart for study screening and selection.

The initial electronic database search identified 184 studies (108 from PubMed and 76 from Scopus), of which 69 were duplicates. The updated search on 21 October identified another 25 articles from PubMed, giving us a total of 209 studies. A total of 140 titles and abstracts were screened after duplicates were removed, out of which 45 were considered eligible for full text screening. Six studies were further excluded; five of them did not report on the outcomes of interest and one had no full text available. A total of 39 studies were included in the final qualitative analysis. The flow and screening process are described in detail in Figure 1.

### 2.2. Characteristics of Studies

An overall of 3651 samples, from 2825 patients and 145 controls, were extracted from the 39 included studies. The studies were conducted worldwide; 16 in Asia [12,19,20,21,22,23,24,25,26,27,28,29,30,31,32,33], 14 in Europe [34,35,36,37,38,39,40,41,42,43,44,45,46,47], and 9 in North America [48,49,50,51,52,53,54,55,56]. A minority of studies (8) included control samples [21,28,29,33,38,47,54,56]. One study did not report the number of samples or patients studied [26].

The sources of samples were heterogenous among the included studies. The most used source was the nasopharyngeal swabs. Other commonly used sources were the blood, plasma, saliva, oropharyngeal, sputum, and respiratory excretions. Some studies also reported the use of rectal, urine, and bronchoalveolar lavage (BAL) samples [30,44,45]. One study reported using colostrum samples [55]. Table 1 and Table 2 summarize the characteristics and main findings of the articles included.

### 2.3. Diagnostic Performance of Digital PCR

Evidence suggests that ddPCR assays exhibit an enhanced analytical sensitivity, as assessed by measuring their limit of detection (LOD). Abasiyanik et al., reported LODs of 0.06 and 0.21 copies/µL, while Kim et al. 1.99 (95% CI: 1.38–3.38) and 5.18 copies/µL (95% CI: 3.33–12.93), respectively, while targeting the N1 and N2 genes [32,48]. Two other groups designed their ddPCR assays targeting the ORF1ab and N genes; Dong et al. measured LODs of 2 copies/reaction for both genes, whereas Nyaruaba et al. 1.42 and 2.75 copies/reaction for the ORF1ab and N genes, respectively [22,27]. Of interest, another study targeted the E, N, RdRP, N2, and N3 genes, reporting LODs ranging from 7 to 24.3 copies/reaction [42]. Notably, the LODs of ddPCR assays were found to be lower than those of RT-qPCR, meaning that ddPCR showed a higher analytical sensitivity. Zhang et al. targeted the ORF1ab and N genes, measuring LODs of 401.8 (95% CI: 284.8–938.3) and 336.8 (95% CI: 244.6–792.5) copies/mL for ddPCR, whereas 520.1 (95% confidence interval (CI): 363.23–1145.69) and 528.1 copies/mL (95% CI: 347.7–1248.7) for RT-qPCR [31]. Martin et al. reported a LOD of 77 copies/mL (8 copies/reaction) for their ddPCR, in contrast to 170 copies/mL (34 copies/reaction) for their reference RT-PCR assay [41], while Alteri et al. found a LOD of 2.9 copies/reaction (95% CI: 2.0–11.5), which was 5 to 10 times lower that the LODs of the comparison RT-qPCR assays used [34]. Of interest, one study found striking differences among the LODs reported between the ddPCR and reference RT-qPCR assays tested, while targeting the ORF1ab and N genes. Whereas the LODs of ddPCR for the two genes were 2.1 (95% CI: 1.5–4.2) and 1.8 (95% CI: 1.4–3.3) copies/reaction, they were 1039 (95% CI: 763.2–1862) and 873.2 (95% CI: 639.8–1633.2) copies/reaction for RT-qPCR, translating to an approximate 500 times higher analytical sensitivity of ddPCR [12].

Besides its enhanced analytical performance, all included studies revealed that ddPCR had at least a comparable clinical performance with RT-qPCR, while most reported that ddPCR exhibited a superior detection ability. The main findings are summarized in Table 1. Several studies showed that ddPCR was able to detect the SARS-CoV-2 virus in samples found negative with RT-qPCR, especially when the viral load copy number was low. For instance, a study performed by Alteri et al. showed that ddPCR was able to detect 19 positive cases with low viral load initially testing negative with RT-qPCR [34]. Cassinari et al. also compared the diagnostic performance of ddPCR with RT-qPCR in saliva and nasopharyngeal swab samples. RT-qPCR was able to detect SARS-CoV-2 in 8/13 saliva samples from patients testing positive with RT-qPCR assay in nasopharyngeal samples, resulting in a sensitivity of 62% [35]. On the other hand, ddPCR was able to detect the SARS-CoV-2 virus in 11/13 of the saliva samples tested, yielding a sensitivity of 85% [35]. Both assays reported concordant results while testing nasopharyngeal swab samples [35]. Moreover, Kim et al. reported that ddPCR was able to detect 63 samples with low copy number of SARS-CoV-2 that were reported as negative by RT-qPCR, of which 55% were from symptomatic patients [32]. The improved detection ability of ddPCR was tested in different populations and samples. A study by Ramirez-Rosas et al. showed that ddPCR detected more positive SARS-CoV-2 samples compared with RT-qPCR in nasopharyngeal samples from mothers (41/133 compared to 16/133, respectively) and neonates (24/131 compared to 11/131, respectively), also in colostrum samples (20/140 compared to 3/140, respectively) [55].

Of interest, a study conducted by Dong et al. reported that the true positive rate of detection was drastically improved from 28.2% to 87.4% upon testing with ddPCR compared to digital PCR in patients with fever [22]. Moreover, digital PCR was able to resolve a few ambiguous RT-qPCR results, detecting the SARS-CoV-2 virus in 17 cases of SARS-CoV-2 reported as equivocal (16/17) or negative (1/17) from 29 close contacts [22]. Marchio et al. also demonstrated the ability of ddPCR to clarify RT-qPCR ambiguous cases, reporting as positive 2/8 equivocal samples [40].

A reason why ddPCR exhibits an enhanced SARS-CoV-2 detection ability compared to RT-qPCR was shown in a study by Szwebel et al.; the authors reported that ddPCR displayed increased tolerance to PCR inhibitors, in contrast to RT-qPCR, and revealed its emerging potential for viral diagnostics [45].

Whereas most studies contrasted the performance of ddPCR with RT-qPCR, a few used a different comparison tool. For instance, a study compared ddPCR with LumiraDx rapid antigen testing, with the former showing superior diagnostic accuracy by detecting false-negative results of the latter [36].

### 2.4. Prognostic and Therapeutic Value of Digital PCR

Accumulating evidence suggests that the dynamic monitoring of the SARS-CoV2 RNA plasma levels correlate with the disease status (progression or remission) and response to therapy. Although most eligible studies of this systematic review evaluated the diagnostic performance of ddPCR compared to RT-qPCR, we also extracted data from 10 studies assessing the prognostic performance of ddPCR and their main findings are summarized in Table 2. Four of them showed that ddPCR can accurately quantify SARS-CoV-2 RNA levels, especially in immunocompromised patients and hospitalized patients with severe COVID-19 infections [23,37,46,49]. Furthermore, a study conducted by Ram-Mohan et al. showed that ddPCR, compared to traditional RT-qPCR, was superior for detecting and quantifying SARS-CoV-2 RNAemia in hospitalized patients over a course of 30 days [52]. ddPCR was still able to detect RNAemia in 6.8% of patients at seven days, whereas RT-qPCR could not detect any RNAemia after three days [52]. Notably, this study showed that the higher levels of SARS-CoV-2 RNA in the patients’ plasma were correlated with a higher probability of developing severe disease [52].

A study by Szwebel et al. revealed that ddPCR was able to predict the clinical deterioration of a patient by detecting elevated SARS-CoV-2 RNA plasma levels [45]. Similarly, the virus clearance from the plasma was associated with the patient’s clinical recovery [45]. This indicates that ddPCR may be used to monitor the course of COVID-19 disease. A study by Veyer et al. further corroborated these findings, showing that ddPCR was able to detect SARS-CoV RNAemia in 74% of the hospitalized pneumonia patients tested, while the presence of RNAemia at presentation was associated with disease severity, longer hospital length, progression, and extrapulmonary complications [47].

A few studies have also highlighted the role of ddPCR in predicting response to COVID-19 therapy [30,43]. Sabbatinelli et al. revealed that low serum miR-146a-5p levels, detected with ddPCR, were associated with resistance to treatment with the anti-IL-6 receptor tocilizumab (TCZ) and poor overall survival [43]. Lastly, Yu et al. found that the viral load was markedly higher in early and progressive stages of the disease compared with the recovery stage—thus, higher viral loads may indicate disease progression—suggesting that an accurate quantification via ddPCR at different time points may be the key to monitor the disease course and evaluate response to therapy [30].

## 3. Discussion

**Figure 2:**
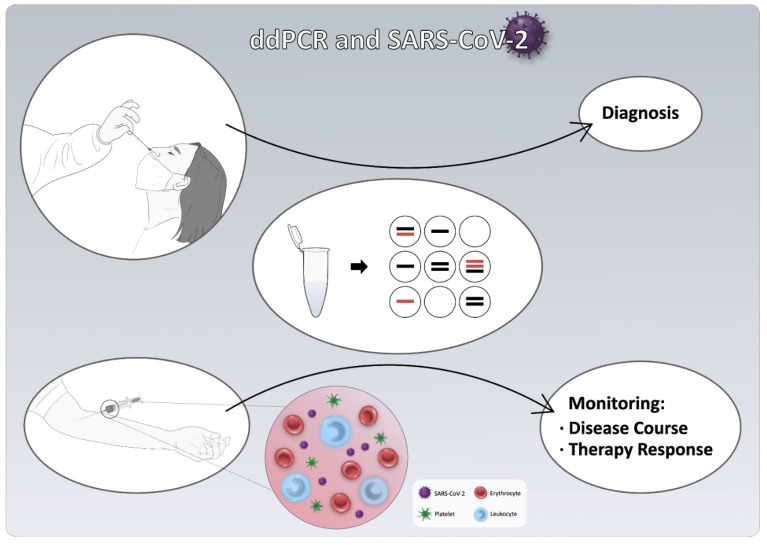
Diagnostic, prognostic, and therapeutic value of droplet digital PCR (ddPCR) in COVID-19 patients.

This systematic review suggests ddPCR is a high-performance modality of potential diagnostic, prognostic, and therapeutic value in COVID-19 patients (Figure 2). ddPCR’s enhanced diagnostic potential was shown in a recent meta-analysis, exhibiting a higher sensitivity than RT-qPCR and LAMP in detecting SARS-CoV-2; this particular study extracted and analyzed ddPCR data from seven studies [57]. Our findings also indicate that the diagnostic performance of ddPCR was at least comparable with RT-qPCR, with the vast majority of the included studies showing that ddPCR was superior in detecting and quantifying the SARS-CoV-2 RNA levels. Notably, when the COVID-19 diagnosis is already established, ddPCR’s superb quantifying ability could be used to dynamically monitor the disease course—as high viral RNA levels in plasma imply disease severity or progression, whereas low levels imply remission—in addition to the response to therapy.

Our study demonstrates that ddPCR could aid in decreasing the false-negative or ambiguous results reported with other assays, such as RT-qPCR or rapid antigen tests, especially in samples with low viral loads. Of interest, ddPCR is an end-point system, where the droplets are simply counted as positive (fluorescent) or negative (non-fluorescent). In contrast to RT-qPCR, it provides absolute target quantification (which is based on the amount of positive and negative droplets), without requiring an external calibration curve or keeping the reference material needed to construct it [58,59]. RT-qPCR is susceptible to amplification inhibitors; however, ddPCR is more tolerant to them—due to its nature that involves partitioning of the initial mixture before amplification into multiple reactions, a process that dilutes the inhibitors and enhances the signal/noise proportion—resulting in higher SARS-CoV-2 detection rates and enhanced reproducibility [45]. As a result, it has a much lower LOD [56,60]. For instance, one study conducted by Martin et al. measured the LODs of RT-PCR and ddPCR, revealing that ddPCR’s LOD was 8 copies/reaction, in contrast to RT-PCR’s 34 copies/reaction [41]. RT-qPCR’s lower analytical sensitivity could explain its higher false negative reports [21,61].

Because of its excellent detection ability, ddPCR could be a valuable assay to complement, or even replace RT-qPCR under specific scenarios. For instance, it could be the primary testing method in the hospital setting, especially in samples of low or fluctuating viral loads, or when the clinical suspicion is high. Furthermore, ddPCR could aid in resolving the undetermined results reported by RT-qPCR or in confirming negative results of patients before their discharge, leading to improved surveillance and control and reduction of false-negative interpretations [40]. In the community setting, ddPCR could be useful for the early detection of SARS-CoV-2, especially when RT-qPCR testing is negative in individuals at risk of carrying the virus, shortening the window period. Of interest, there is evidence that the SARS-CoV-2 virus appears in the saliva in the early course of the infection [53]. A study conducted by Cassinari et al. showed that ddPCR had superior sensitivity compared to RT-qPCR (85% versus 62%) when testing saliva samples from patients positive for SARS-CoV-2 with nasopharyngeal sampling [35].

As mentioned before, ddPCR has several advantages over other commercial tests used for COVID-19 diagnostics, such as RT-qPCR. However, because of certain disadvantages—such as its limited availability, higher cost, relatively low throughput, and longer turnaround times compared to RT-qPCR—it could be unfeasible to regularly perform ddPCR in the community setting, instead of rapid antigen or RT-qPCR testing [34,40]. Some of these disadvantages can yet be overcome. For instance, Yin et al. designed a rapid digital PCR providing robust results in less than 15 min [29]. Similar to other assays used for SARS-CoV-2 detection, another drawback of ddPCR includes the generation of false positive results, caused by the inaccurate interpretation of negative droplets as positive [58]. The presence of false positive droplets could even appear while testing no-template controls [58]. To deal with this issue, a few of the included studies focused their investigation on symptomatic patients, for example during their hospitalization [21,22,34,48]. This helped confirm their clinical suspicion and our findings indicate the ddPCR outperformed RT-qPCR in such clinical scenarios. One way to deal with potential false positive results in asymptomatic patients could be to retest a sample using the same or a different assay. Notably, a study by Yin et al. showed that RT-qPCR required more undetermined samples to be retested, when compared to ddPCR [29].

Multiple parameters need to be adjusted, as they could impact the sensitivity of ddPCR. These include the amount of input genetic material, the concentration of the primers used in the reaction, the cycling conditions (e.g., duration of denaturation, annealing, and extension; number of cycles; temperature during annealing and extension), also determining the fluorescence threshold and LOD [62]. Of interest, a study showed that undigested fragments could fail packaging into droplets, compromising the performance of the assay [63]. Even if less likely than RT-qPCR, ddPCR detection ability could also be impacted by inhibitors or non-target genetic material. Thus, optimization of the ddPCR assay and strict quality control measures are needed to achieve accurate and reproducible results [58,62]. Improved artificial intelligence algorithms could also help towards this direction [64].

Apart from its enhanced diagnostic performance, ddPCR could play a key role in stratifying disease severity, assessing prognosis, and monitoring response to therapy in COVID-19 patients. Although evidence is still preliminary, ddPCR may have the potential to become an ancillary tool or the primary modality in these settings. One study reported elevated SARS-CoV-2 RNA concentration in plasma was associated with severe illness in COVID-19 patients [49]. Furthermore, a higher viral load in the plasma was associated with increased mortality or ICU admission [46,49]. ddPCR was also accurate in detecting and quantifying SARS-CoV-2 RNA plasma levels in immunocompromised and ICU patients, a finding with potential clinical significance [23,37,46,49]. Indeed, ddPCR could serve as the emerging diagnostic modality of choice to monitor patients under such settings. In addition, evidence has shown that, through quantifying the SARS-CoV-2 RNA plasma levels, ddPCR could monitor COVID-19 patients’ disease course and response to therapy [30]. Besides targeting the SARS-CoV-2 genome, ddPCR was further able to assess response to therapy by detecting the levels of serum-miR-146a-5p after treatment with TCZ [43].

Our study mainly compared the diagnostic accuracy of ddPCR with RT-qPCR. Except these two, other NAATs have been used for SARS-CoV-2 detection, for example LAMP. In contrast to PCR-based assays that run in thermal cyclers, the latter operates on a constant temperature (60–65 °C), and its results can be assessed with simple visual detection, without needing a machine [65,66]. LAMP is a fast and economical modality that holds promise in COVID-19 diagnostics. However, its sensitivity has been found to be lower than that of ddPCR [57]. Similar to ddPCR, NGS has also been reported to have diagnostic, prognostic, and therapeutic value in COVID-19 patients, while it has also shown to be robust in identifying coinfections in such patients. NGS comes though with expensive infrastructure and needs highly trained laboratory personnel, including bioinformatics support [67].

This systematic review is not without its limitations. Firstly, there was heterogeneity among the ddPCR and/or RT-qPCR laboratory protocols used in the included studies. There was also a variety of sample sources used, potentially affecting the diagnostic accuracy of ddPCR. A recent study reported that ddPCR’s sensitivity varied among different sources of samples, with the highest positive detection rate being in nasopharyngeal swabs [67], yet further evidence focusing on the diagnostic performance of ddPCR across diverse samples would be desirable. Both protocol and sample source diversity prohibited us from conducting a quantitative analysis. Most of the eligible studies in this review assessed the diagnostic accuracy of ddPCR, especially in comparison to RT-qPCR; in contrast, there were fewer studies that discussed the prognostic value of ddPCR or evaluated its role in monitoring COVID-19 therapy response, further necessitating the need for additional future research. Lastly, limited evidence suggests the distinct SARS-CoV-2 variants do not have a significant impact on the diagnostic performance of NAAT assays and that ddPCR should be effective in detecting them [68,69]. Notably, a study by Perchetti et al. suggested that ddPCR is superior to RT-qPCR for detecting and differentiating COVID-19 variants [70], yet it is clear that more studies are needed to unravel ddPCR’s potential in this scenario.

## 4. Materials and Methods

### 4.1. Search Strategy

This systematic review was conducted according to the standards set by the Preferred Reporting Item for Systematic Review and Meta-Analysis (PRISMA) Statement [71]. Two electronic databases (PubMed and Scopus) were comprehensively searched until 13 July 2021. An additional search was performed on 21 October on the PubMed database. The following search strategy was employed to identity all articles reporting on the diagnostic, prognostic, and therapeutic value of digital PCR in COVID-19 patients: (“digital PCR” OR ddPCR) AND (COVID-19 OR SARSCoV2). No specific filters were used, like publication date or article type.

### 4.2. Study Selection

Articles written in English were considered eligible if they studied or compared the use of digital PCR with other diagnostic techniques in samples derived from humans with a suspected or established COVID-19 diagnosis. There were no restrictions on the type of clinical samples studied (e.g., nasopharyngeal swabs, saliva, and blood) or the phase of COVID-19 infection. Articles were excluded if they studied solely non-human (e.g., cell lines, animal models, environmental) or post-mortem tissue samples. Editorials, reviews, and conference abstracts were also excluded. Duplicates were removed by using the Paperpile reference manager (https://paperpile.com/app, accessed on 13 July 2021).

Two authors (M.M.A. and A.I.) independently performed an initial title and abstract screening using the Rayyan software [72]. This was followed by a full-text screening. Any disagreements were resolved by a third author (I.P.N.). Bibliography lists of all relevant articles were reviewed to identify any studies missed by the database search, using a snowball approach [73].

### 4.3. Data Extraction

Relevant data were extracted independently by two authors (A.I. and M.M.A.) on an Excel^®^ spreadsheet. Any disagreements were resolved by a third author (I.P.N.). The following data were extracted: first author, year, number of samples, number of patients and/or controls, source of samples, diagnostic performance of ddPCR and/or comparison methodology, and main ddPCR findings of potential prognostic and therapeutic value.

### 4.4. Study Outcomes

This study aimed to assess the diagnostic, prognostic, and therapeutic value of ddPCR in COVID-19 patients, regardless of the sample type. It also aimed to compare the diagnostic performance of ddPCR with other diagnostic tools such as RT-qPCR and rapid antigen testing.

## 5. Conclusions

Current evidence suggests that ddPCR may improve SARS-CoV-2 detection, besides monitoring the disease course and treatment response of COVID-19 patients. This review indicates that ddPCR diagnostic ability could be superior to that of other diagnostic methods, including RT-qPCR and rapid antigen testing. Additionally, our findings suggest that ddPCR is a robust prognostic tool in COVID-19 patients, as it can effectively quantify and monitor the SARS-CoV-2 viral load levels throughout the course of the disease. Thus, it could serve as the modality of choice in selected clinical scenarios, such as in monitoring hospitalized patients and confirming their negative results before discharge, also in resolving indeterminate RT-qPCRs. However, further research—especially in the form of prospective studies and randomized clinical trials—is needed to standardize its validity and clinical utility and fully decipher its value.

## Figures and Tables

**Table 1:** Diagnostic performance of droplet digital PCR (ddPCR) compared to traditional testing methods in the included studies.

First Author (Year)	Number of Patients (Samples)		Sources of Samples	Summary of Results
	Patients (Samples)	Controls		
Abasiyanik et al. (2020) [48]	166	N/A	Nasopharyngeal swabs Saliva	ddPCR showed higher sensitivity than RT-qPCR, especially in low viral load samples. ddPCR detected eight more positive cases than RT-qPCR.
Alteri et al. (2020) [34]	55 (100)	N/A	Nasopharyngeal swabs	ddPCR was positive in 19/55 cases tested negative with RT-qPCR, all of which had a low viral load.
Cassinari et al. (2021) [35]	130	N/A	Nasopharyngeal swabs Saliva	ddPCR was more sensitive than RT-qPCR (85% and 62%, respectively) with saliva testing, whereas both modalities showed concordance with nasopharyngeal testing.
Cento et al. (2021) [36]	960 (960)	N/A	Nasopharyngeal swabs	ddPCR was positive in 50 samples with a low viral load deemed negative by the LumiraDx rapid antigen test.
Chen et al. (2021) [20]	52 (87)	N/A	Blood Oropharyngeal swabs	ddPCR exhibited higher sensitivity and specificity than RT-qPCR, especially in blood samples with a low viral load.
Dang et al. (2020) [21]	30 (117)	61	Pharyngeal swabs Sputum	Positive detection rate of ddPCR was 100%, compared with 93.3% of RT-qPCR. Three samples negative with RT-qPCR were tested positive with ddPCR. 17 samples of a low viral load were tested positive with ddPCR, yet only 9/17 were positive with RT-qPCR.
de Kock et al. (2020) [38]	5 samples	5 samples	Nasopharyngeal swabs	Sensitivity of RT-qPCR decreased in the presence of background nucleic acids, in contrast to ddPCR’s.
Dong et al. (2021) [22]	103 (196)	N/A	Pharyngeal swabs	From the 103 febrile patients included in this study, ddPCR was positive in 19/25 negative and 42/49 equivocal RT-qPCR results; sensitivity was improved from 28.2% to 87.4%. From 29 close contacts, ddPCR detected SARS-CoV-2 in 16 equivocal and 1 negative RT-qPCR results.
Duong et al. (2021) [56]	20 (60)	12	Nasopharyngeal swabs	ddPCR showed higher sensitivity and precision with a lower LoD compared to RT-qPCR.
Falzone et al. (2020) [39]	1 (multiple dilutions)	1 (multiple dilutions)	Nasopharyngeal swabs	ddPCR showed enhanced detection ability than RT-qPCR in diluted samples with a low viral load.
Gniazdowski et al. (2020) [50]	185	N/A	Nasopharyngeal swabs	ddPCR showed enhanced SARS-CoV-2 detection ability compared to RT-qPCR. ddPCR assay was positive in low viral load samples tested negative with RT-qPCR.
Jiang et al. (2020) [24]	10 (32)	N/A	Nasopharyngeal swabs Oropharyngeal swabs Blood	ddPCR showed enhanced SARS-CoV-2 detection ability compared to RT-qPCR. Whereas all RT-qPCR positive samples were also ddPCR positive, three RT-qPCR negative clinical samples were tested positive with ddPCR.
Kim et al. (2021) [32]	366 samples	N/A	Nasopharyngeal swabs Sputum samples Blood samples	63 samples negative with RT-qPCR were positive with ddPCR. These 63 samples had low copy numbers, while only 55% of them were from symptomatic patients.
Lee et al. (2021) [33]	20	20	Pharyngeal swabs Sputum samples	ddPCR required a lower sample concentration compared to RT-qPCR to detect SARS-CoV-2.
Liu et al. (2020) [25]	43 (74)	N/A	Stool samples Sputum samples Throat swabs	Whereas RT-qPCR was negative in 18 samples received from 9 relapsed patients, ddPCR was positive in 12 of them. ddPCR performed better than RT-qPCR in samples of a low viral load. ddPCR had a positive detection rate of 55.41%, compared to RT-qPCR’s 36.5%.
Liu et al. (2020) [26] *	N/A	N/A	Nasopharyngeal swabs	ddPCR was positive in samples of a low viral load testing negative with RT-qPCR. ddPCR showed higher sensitivity and precision than RT-qPCR.
Marchio et al. (2021) [40]	208 samples	N/A	Nasopharyngeal swabs	8.6% of the negative RT-qPCR results were deemed positive with ddPCR. All positive samples detected by RT-qPCR were confirmed by ddPCR. ddPCR was positive in two samples with ambiguous RT-qPCR results.
Martin et al. (2021) [41]	448 samples	N/A	Nasopharyngeal swabs	ddPCR’s detection performance in group testing was comparable with RT-qPCR’s, yet the former showed a lower LoD.
Mio et al. (2021) [42]	90	N/A	Nasopharyngeal swabs	ddPCR’s detection performance was comparable with RT-qPCR’s, yet the former performed better at low concentrations.
Nyaruaba et al. (2020) [27]	94 samples	N/A	Oropharyngeal swabs	ddPCR was more sensitive than RT-qPCR (96.3% versus 92.6%).
Park et al. (2021) [19]	5 (8)	N/A	Nasopharyngeal swabs Oropharyngeal swabs	ddPCR’s detection ability was at least equivalent to RT-qPCR’s; yet, unlike RT-qPCR, ddPCR’s performance was not affected by the primer-probe sets’ sequences.
Poggio et al. (2021) [51]	64	N/A	Nasopharyngeal swabs	Out of the 18 RT-qPCR negative patients, 11 tested positive with ddPCR.
Ramirez-Rosas et al. (2021) [55]	404 samples	N/A	Nasopharyngeal swabs Colostrum samples	Samples from asymptomatic mothers and their neonates were tested. ddPCR detected 25 more positive samples (41/133) than RT-qPCR (16/133) from the mothers’ nasopharyngeal swabs tested. ddPCR detected 13 more positive samples (24/131) than RT-qPCR (11/131) from the neonates’ nasopharyngeal swabs tested. ddPCR detected 17 more positive samples (20/140) than RT-qPCR (3/140) from the colostrum samples tested.
Savela et al. (2021) [53]	7 (105)	N/A	Saliva Nasopharyngeal swabs	Saliva was superior to nasopharyngeal sampling for the early detection of SARS-CoV-2. ddPCR performed better than RT-qPCR in the saliva testing.
Scutari et al. (2020) [44]	2	N/A	Nasopharyngeal swabs Rectal swabs Urine Bile Plasma	ddPCR allows SARS-CoV-2 quantification in multiple sample types and even many days after the onset of symptoms In one patient and unlike RT-qPCR, ddPCR on nasopharyngeal swabs detected the SARS-CoV-2 RNA at different time points during hospitalization.
Sun et al. (2021) [28]	21	6	Throat swabs Sputum swabs Anal swabs	ddPCR showed higher sensitivity compared to RT-qPCR, especially at low viral load samples.
Suo et al. (2020) [12]	77	N/A	Throat swabs	ddPCR showed higher sensitivity [94% (95% CI: 83–99%), vs. 40% (95% CI: 27–55%)] and NPV [63% (95% CI: 33–83%) vs. 16% (95% CI: 13–19%)] compared to RT-qPCR.
Szwebel et al. (2021) [45]	1	N/A	Nasopharyngeal swabs BAL Plasma	ddPCR displayed increased tolerance to PCR inhibitors, showing high potential for viral diagnostics.
Tedim et al. (2021) [46]	90	N/A	Nasopharyngeal swabs Plasma	ddPCR detected the viral RNA in the plasma of 36 patients, whereas RT-qPCR was positive in 34 (94.4%) of them.
Xu et al. (2021) [54]	30 samples	30 samples	Nasopharyngeal swabs	ddPCR had higher sensitivity and specificity compared to RT-qPCR, especially in samples with low viral load. ddPCR had a PPV of 97.9%.
Yin et al. (2021) [29]	6	3	Throat swabs	Authors developed a rapid ddPCR which yielded robust results within 15 min By testing serial diluted samples, rapid ddPCR was accurate at both positive and negative reference samples; it was also more consistent than RT-qPCR at low-viral-load sample testing.
Yu et al. (2020) [30]	76 (323)	N/A	Nasal swabs Throat swabs Sputum Blood Urine	Of the 161 negative samples reported by RT-qPCR, 4 (4/161) were positive with ddPCR.
Zhang et al. (2020) [31]	24 (34)	N/A	Throat swabs Anal swabs Sputum Blood	ddPCR was more accurate than RT-qPCR in detecting positive samples with low viral load. Positive rates were higher in ddPCR (67.7%) than in RT-qPCR (58.8%).

* This study does not specify the number of samples tested from patients and healthy controls. Abbreviations: ddPCR, droplet digital polymerase chain reaction; dPCR, digital polymerase chain reaction; RT-qPCR, reverse transcription quantitative polymerase chain reaction; SARS-CoV-2, severe acute respiratory syndrome coronavirus 2; NPV, negative predictive value; PPV, positive predictive value; RNA, ribonucleic acid; BAL, bronchoalveolar lavage; LoD, limit of detection, N/A: not available, CI: confidence interval.

**Table 2:** Prognostic and therapeutic value of droplet digital PCR (ddPCR) in COVID-19 patients.

Author (Year)	Number of Samples		Source of Sample	Summary of Results
	Patients (Samples)	Controls		
Bermejo-Martin et al. (2020) [49]	250	N/A	Plasma	ddPCR offered robust detection and quantification of SARS-CoV-2 RNA in the patients tested. SARS-CoV-2 RNAemia was detected in most ICU patients (78%), in contrast to ward patients (27%) or outpatients (2%). High SARS-CoV-2 RNA levels in the plasma were significantly correlated with disease severity.
Chen et al. (2021) [20]	52 (87)	N/A	Plasma Oropharyngeal swabs	High viral RNA levels in the plasma were more common in critical than general or severe patients. Monitoring with ddPCR showed that critical patients were not able to clear the viral load in the plasma, in contrast to general and severe patients. Elevation of viral RNA levels in the plasma was associated with disease progression.
Colagrossi et al. (2021) [37]	41	N/A	Nasopharyngeal swabs BAL Plasma	ddPCR precisely quantified the SARS-CoV-2 RNA in immunocompromised patients and patients with severe infection. SARS-CoV-2 RNAemia was associated with high viral levels in the respiratory samples, presence of hematological malignancies, and poor OS.
Hu et al. (2020) [23]	47	N/A	Throat swabs Deep sputum	ddPCR was able to precisely quantify SARS-CoV-2 in hospitalized patients.
Ram-Mohan et al. (2021) [52]	191	N/A	Nasopharyngeal swabs Plasma	ddPCR was more robust than RT-qPCR for the detection of viral RNAemia and disease monitoring. Baseline RNAemia was detected in 23% (44/191) of the patients with ddPCR, versus in 1.4% (2/147) of them with RT-qPCR. On the third and seventh day, ddPCR detected viral RNAaemia in 13% (6/45) and 6.8% (3/44) of the specimens, whereas RT-qPCR was negative On the 30th day, both ddPCR and RT-qPCR were negative for all samples tested. Baseline RNAemia was associated with disease severity, longer hospitalization, progression, and extrapulmonary complications.
Sabbatinelli et al. (2021) [43]	30	N/A	Serum	Low serum miR-146a-5p levels, detected with ddPCR, were associated with resistance to treatment with the anti-IL-6 receptor TCZ and poor OS.
Szwebel et al. (2021) [45]	1	N/A	Nasopharyngeal swabs BAL Plasma	Monitoring with ddPCR showed an increase of SARS-CoV-2 plasma levels preceded the patient’s clinical deterioration, while the virus clearance was associated with full recovery.
Tedim et al. (2021) [46]	90	N/A	Nasopharyngeal swabs Plasma	SARS-CoV-2 RNAemia detected either with ddPCR was much more prevalent in ICU (91%), rather than ward patients (27%) or outpatients (23%). SARS-CoV-2 RNAemia was associated with disease severity.
Veyer et al. (2020) [47]	58	12	Plasma	SARS-CoV-2 RNAemia, detected with ddPCR, was associated with disease severity and clinical deterioration.
Yu et al. (2020) [30]	76 (323)	N/A	Nasal swabs Throat swabs Sputum Blood Urine	While performing ddPCR on sputum samples, patients in the early or progressive stage exhibited significantly higher SARS-CoV-2 RNA levels than the ones in the recovery stage of the disease. ddPCR was important to assess response to therapy through quantifying the viral load.

Abbreviations: ddPCR, droplet digital polymerase chain reaction; RT-qPCR, real time quantitative polymerase chain reaction; SARS-CoV-2, severe acute respiratory syndrome coronavirus 2; TCZ, tocilizumab; RNA, ribonucleic acid; BAL, bronchoalveolar lavage; OS, overall survival.

## Data Availability

Data is contained within the article.
